# Dynamics of Fluoride Bioavailability in the Biofilms of Different Oral Surfaces after Amine Fluoride and Sodium Fluoride Application

**DOI:** 10.1038/srep18729

**Published:** 2016-01-05

**Authors:** Ella A. Naumova, Christoph Dickten, Rico Jung, Florian Krauss, Henrik Rübesamen, Katharina Schmütsch, Tudor Sandulescu, Stefan Zimmer, Wolfgang H. Arnold

**Affiliations:** 1 Dept. of Biological and Material Sciences in Dentistry, Faculty of Health, School of Dentistry, Witten/Herdecke University, Witten, Germany; 2 Dept. of Operative and Preventive Dentistry, Faculty of Health, School of Dentistry, Witten/Herdecke University, Witten, Germany

## Abstract

It was the aim of this study to investigate differences in fluoride bioavailability in different oral areas after the application of amine fluoride (AmF) and sodium fluoride (NaF). The null hypothesis suggested no differences in the fluoride bioavailability. The tongue coating was removed and biofilm samples from the palate, oral floor and cheeks were collected. All subjects brushed their teeth with toothpaste containing AmF or NaF. Specimens were collected before, as well as immediately after and at 30 and 120 minutes after tooth brushing. The fluoride concentration was determined. The area under the curve was calculated for each location and compared statistically. In the tongue coating, fluoride concentration increased faster after NaF application than after AmF application. After 30 minutes, the fluoride concentration decreased and remained stable until 120 minutes after AmF application and returned to baseline after NaF application. The difference between the baseline and the endpoint measurements was statistically significant. The fluoride concentration in the tongue coating remained at a higher level compared with the baseline for up to 120 minutes post-brushing. This may indicate that the tongue coating is a major reservoir for fluoride bioavailability. The results also indicate an unequal fluoride distribution in the oral cavity.

The oral cavity is a unique environment with different niches that house various microbial species in their covering biofilms^1^. The oral biofilm has several functions: It acts as an interface between the oral surface and its surroundings; it harbors bacteria and interacts with other bacteria; it provides a mucosal protective coating for mechanical and antimicrobial defense^2^.

Many factors have an impact on the biofilm formation, including host genetics, ethnicity, smoking, diet and its impact on the local pH^2^, local temperature in different sites of the oral cavity^3^, age, environment, sexual behavior^4^, and hormonal level^5^. Still other factors are pharmacological influences^6^, oral health^7^,^8^,^9^,^10^, epithelial morphology^11^, the content of saliva and its rheological and clearance properties^1^,^11^,^12^,^13^, and the use of oral hygiene products^14^. Biofilm formation is regulated by complex biological mechanisms, which include host–microbial cross talk^1^,^2^,^11^,^12^,^13^, epithelial cell signaling^15^, and quorum-sensing^16^. At the beginning of biofilm formation and bacterial colonization, the surface components of the bacteria adhere to oral epithelial cells^15^. The material properties to which biofilms adhere have an impact on biofilm formation^17^ and there is no uniform epithelial lining of the oral cavity. Morphologically, the oral mucosa is divided into the lining mucosa, masticatory mucosa, and specialized mucosa^18^. These epithelia differ in their thickness and surface texture^19^,^20^. Another study has shown that the lining epithelial surface morphology varied in the different areas of the oral cavity and that this surface morphology influenced the composition of the overlying biofilm^11^. The oral microflora composition varies on distinct oral surfaces (e.g., teeth, mucosa), and at specific sites (e.g., fissures, gingival crevice)^21^. From a bacteriological point of view, seven different locations in the oral cavity are distinguished by their varying microbiota^22^. These are located in the different oral niches with various origins, composition, and structural and functional peculiarities^2^,^23^.

Because of the differences in epithelial surface textures, the rheological properties of saliva and the composition of the oral biofilms in the various niches, the dynamics and distribution of proteins, polysaccharides and electrolytes such as fluoride may also vary considerably.

It is now widely accepted that fluorides are the most effective agents in caries prevention^24^,^25^. Fluorides are administered mainly through toothpaste. Other forms of application are fluoridated water^24^, fluoridated salt^26^ or milk^27^,^28^, fluoridated mouth rinses^29^ and fluoride varnishes^30^. After application, fluorides are distributed within the oral cavity and remain in saliva for a certain period of time facilitating the remineralization of dental enamel^31^,^32^,^33^. However, there is a growing concern about possible side effects of fluoride administration. High doses of systemic fluoride administration in early childhood are causing fluorosis, which results in mineralization defects of dentin and enamel^34^.

Therefore, individualized regimes for fluoride application in caries prevention in young children should be elaborated. To achieve this goal, more detailed knowledge about the dynamics of fluoride distribution after its application into the oral cavity is necessary. Detailed knowledge about the dynamics of the fluoride kinetics in saliva after fluoride application is already available^35^,^36^,^37^. It has also been shown that fluoride distribution in saliva is different in supernatant saliva, salivary sediment and in plaque^32^,^33^,^37^,^38^,^39^. However, not much is known about the fluoride distribution after tooth brushing in the biofilm of different oral compartments. It was therefore the aim of this study to investigate the dynamic distribution of fluoride in different oral niches after the application of two different fluoride compounds.

The primary research question of this investigation was whether there are differences between the fluoride concentrations in different biofilms of the oral mucosa over a time period of 120 minutes after application of amine fluoride (AmF) or sodium fluoride (NaF). The secondary aims were to determine the variation in the fluoride concentration in two ways: between the different collection times and between both fluoride compounds at the different collection times.

The null hypothesis suggested that there are no differences in the fluoride bioavailability in all investigated oral areas and between the two tested fluoride compounds.

## Materials and Methods

### Subjects

Thirty-two healthy dental students (16 men and 16 women) in their first, second and third years of education (mean age: 24.06 ± 2.17 years; body weight: 69.7 ± 13.5 kg) were recruited and randomly evenly distributed into two groups of eight males and eight female subjects. All subjects received verbal and written information about the investigation, and written instructions regarding the schedule of the study design and the proper tooth brushing method. The subjects provided their informed consent. Inclusion criteria were satisfactory oral and general health. The exclusion criterion was the presence of active caries or periodontal disease. All participants of this study were allowed to retain to their daily tooth brushing protocol including their custom toothpaste in the morning and evening. They did not drink fluoridated water or took any other fluoridated aliments during the experimental period. This protocol was approved by the ethical committee of Witten/Herdecke University (permission 25/2013). All experiments were performed in accordance with relevant guidelines and regulations.

### Study design

The oral biofilm samples were collected between 2:00 and 4:30 p.m. (in the spring and summer time). At 2:00 p.m., the experiment started with the collection of baseline samples (T0). The subjects in group 1 were asked to brush their teeth with toothpaste containing an amine fluoride (AmF, 1400 pm fluoride, Elmex, CP GABA GmbH, Hamburg, Germany). The subjects in group 2 brushed their teeth with toothpaste containing a sodium fluoride (NaF, 1400 ppm fluoride, Eurodont, Maxim Markenprodukte, Pulheim, Germany). Further samples were collected immediately (T1), 30 min (T2) and 120 min (T3) after tooth brushing.

### Tooth brushing and collection of saliva, tongue coating and oral biofilm samples

**Figure 1:**
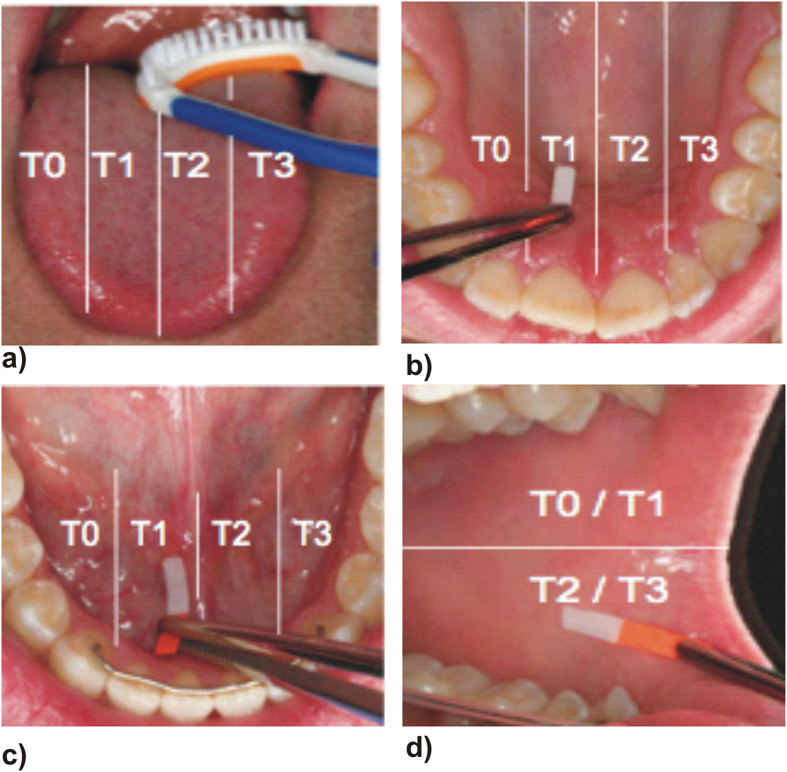
Collection areas in the different oral niches: (a) tongue, (b) palate, (c) mouth floor and (d) cheeks.

**Figure 2:**
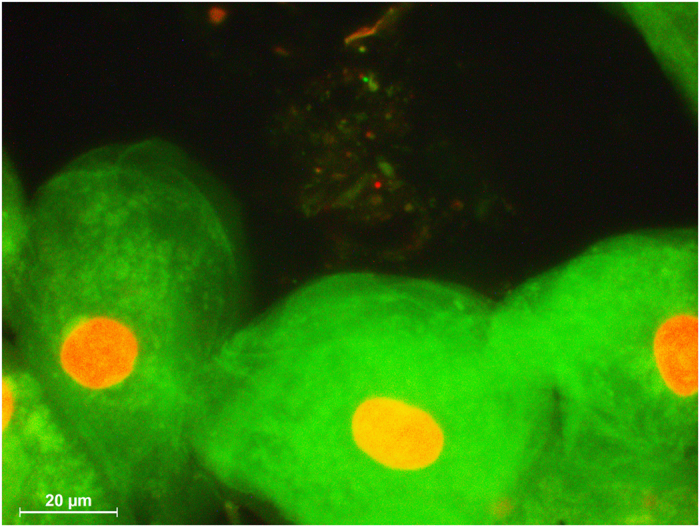
Fluorescence microphotograph of the biofilm of the cheek surface collected with periopaper and stained with life – dead bacterial staining. The smear contains epithelial cell at the bottom and life (green) and dead (red) bacteria.

Each subject underwent the experimental protocol five times. For standardization, all subjects brushed their teeth with the respective fluoride toothpaste and an Elmex toothbrush using the modified Bass brushing technique^40^ for three minutes. Saliva samples were collected in 20 ml plastic tubes. Immediately after sample collection, 250 μl of TISAB II was added and the samples were frozen and stored at −80 °C until they were assayed for fluoride concentration. To evaluate the fluoride bioavailability dynamics, samples were collected from the tongue, cheeks, oral floor and palate and then divided into four areas: I, II, III and IV (Fig. 1) to avoid double collection of the biofilm in the same area. Tongue coating was collected from four regions of the tongue with the commercially available tongue cleaner (One Drop Only®, Berlin, Germany). Biofilm samples from the palate, oral floor and cheeks were collected for 10 seconds using Periopaper (Oraflow Inc., New York, USA). To ensure the collection of biofilm with periopaper control strips were stained with backlight live/dead staining (Molecular Probes^®^ Invitrogen, Life Technologies, Darmstadt, Germany) according to the manufacturer’s instructions and microscopic pictures were taken with a a fluorescence microscope (Leica DMB, Leica, Wetzlar, Germany) with a N21 (BP 515–560) filter for green fluorescence and a CY5-T (BP 635/10) filter for red fluorescence (Fig. 2).

### Fluoride concentration

The fluoride concentration was determined in all samples according to standard operating procedures (SOP) using a fluoride-sensitive electrode (Orion 96-09, Thermo Electron) as described elsewhere 32.

### Statistical methods

Data were processed with the Statistical Package for Social Sciences (SPSS 22.0, IBM, Armonk, New York, USA). Prior to the experiments, a sample size calculation was performed with a power of 0.8 and a significance level of α < 0.05, which revealed a minimum number of 13 subjects (Axum 7, Mathsoft, Cambridge, Massachusetts, USA). Considering a possible dropout of 20%, 16 men and 16 women test subjects were selected. The data for the sample size calculation were obtained from a previous investigation^41^. The area under the curve (AUC) was calculated for the fluoride concentration from AmF and NaF between T0 and T3 and for the variation of the fluoride concentration, which was determined by calculating the difference between collection times (T1-T0, T2-T1 and T3-T2) for each oral surface. Statistical comparison of the fluoride concentrations between AmF and NaF was performed using the non-parametric Mann-Whitney U test for independent variables. Statistical comparison of the fluoride concentration at the different collection times and between the oral surfaces in the AmF, and the NaF group was performed using the non-parametric sign test with Wilcoxon signed-rank for related variables. The level of significance was p < 0.05.

## Results

### Fluoride measurements

**Figure 3:**
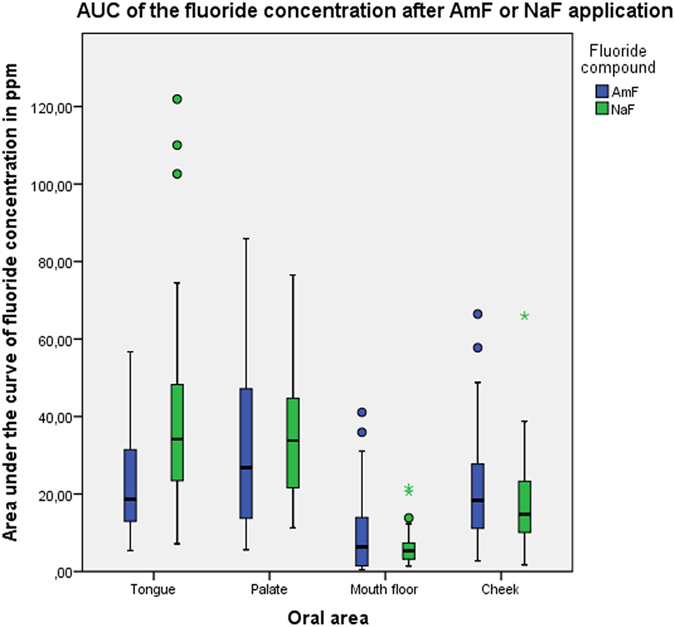
Boxplot graphics of the dynamics of the fluoride concentration in different areas of the oral cavity after AmF and NaF application.

**Figure 4:**
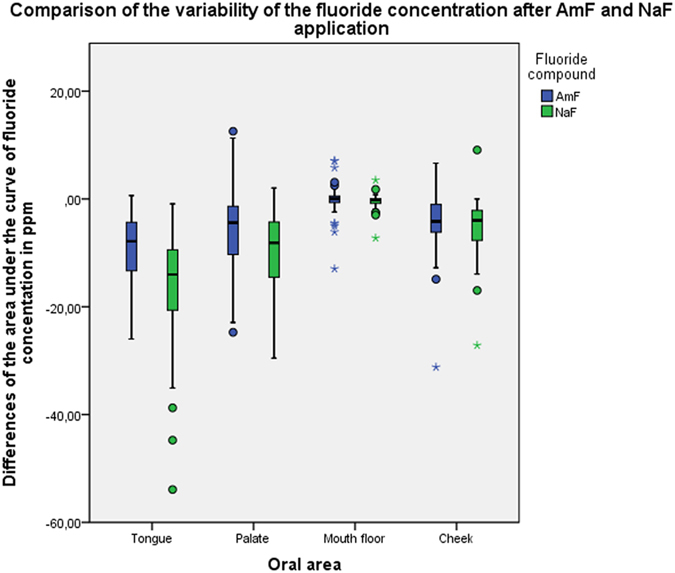
Boxplot graphics of the dynamics of the variation of fluoride concentration after application of AmF and NaF. The AUC has been calculated from the differences between T1-T0, T2-T1 and T3-T2.

A significant difference (p < 0.001) was found for the area under the curve between AmF and NaF samples from the biofilm of the tongue, but not from the biofilms of the other oral areas (Fig. 3). The median of the AUC for the tongue coating fluoride concentration after NaF application was almost twice as high as after AmF application. The descriptive data for the AUC after application of either AmF or NaF are summarized in Table 1 and Table 2, respectively. Comparison of the AUC for the variation of the fluoride concentration between AmF and NaF revealed a significantly higher fluoride concentration decrease after NaF application in the tongue, palate and mouth floor (Fig. 4).

### Fluoride concentrations at the different collection times

At baseline, fluoride concentrations showed significant differences between tongue and palate (p < 0.001), tongue and mouth floor (p = 0.004), tongue and cheek (p = 0.039), palate and mouth floor (p = 0.003) and between palate and cheek (p < 0.001). Comparisons of baseline fluoride concentrations between cheek and mouth floor showed no significant difference (p = 0.858).

**Figure 5:**
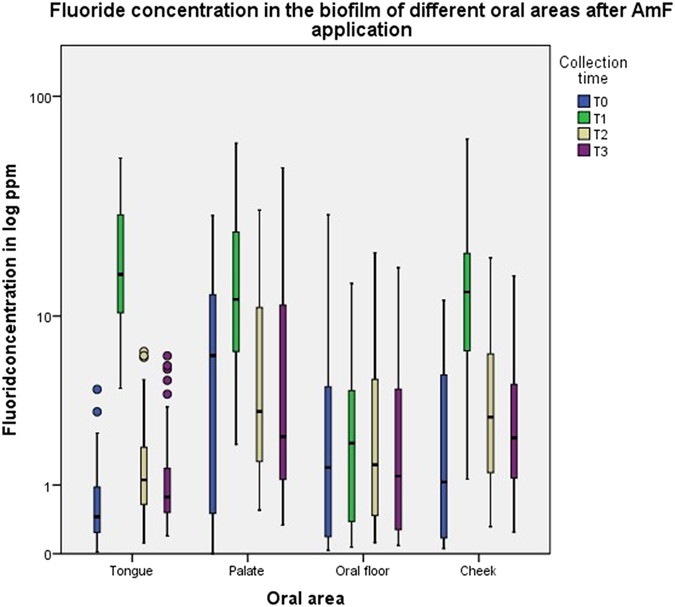
Boxplot graphics of the fluoride concentration at the different collection times after AmF application.

**Figure 6:**
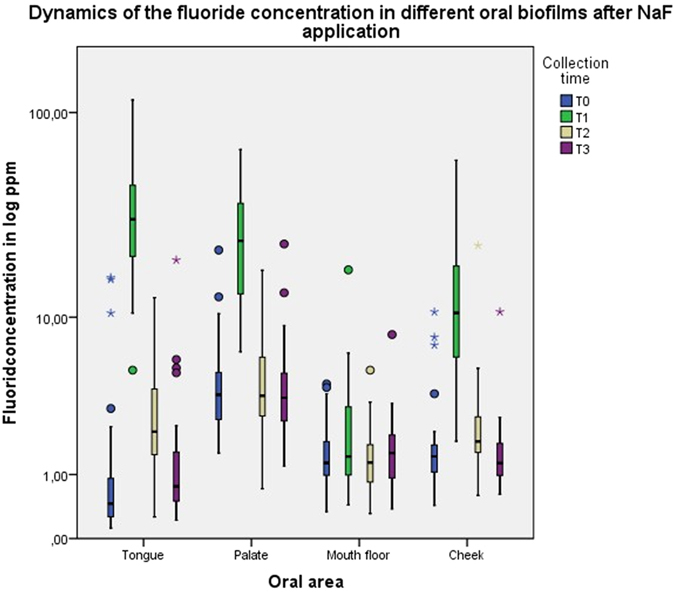
Boxplot graphics of the fluoride concentration at the different collection times after NaF application.

In the tongue coating, the fluoride concentration after tooth brushing with AmF increased to approximately 34-fold of the baseline value (median 0.45 ppm) and stayed about twofold higher than baseline until 120 minutes after the brushing (Fig. 5). After NaF application, the fluoride concentration increased to approximately 68-fold of the baseline (median 0.45 ppm) and decreased to the baseline level after 120 minutes (Fig. 6). In the palate biofilm, the fluoride concentration after tooth brushing with AmF increased twofold above the baseline (median 7.23 ppm) (Fig. 5) and with NaF about six times above the baseline (median 3.71 ppm) (Fig. 6). Fluoride concentration declined to the baseline level 30 minutes after the application of AmF or NaF. In the oral floor biofilm, the fluoride concentration after tooth brushing with AmF increased approximately 0.5-fold above the baseline (median 1.40 ppm) (Fig. 5). After NaF application, no increase of the fluoride concentration was observed (Fig. 6). In the cheek biofilm, the fluoride concentration after tooth brushing with AmF increased above the baseline (median 0.99 ppm) by approximately tenfold and was still twice the baseline value after 120 minutes (Fig. 5). NaF application resulted in a seven-fold increase of the fluoride concentration above the baseline (baseline median 1.35 ppm), which dropped to the baseline level after 120 minutes (Fig. 6).

### Variability of the fluoride concentration

The tongue surface showed the highest variability in fluoride concentration for both fluoride compounds from baseline (T0) to T1 (median 14.69 ppm for AmF and 30.43 ppm for NaF) and from T1 to T2 (median −14.70 ppm for AmF and −28.14 for NaF) followed by palate (T0-T1: median 7.44 ppm for AmF and 19.03 ppm for NaF; T1-T2: −7.34 ppm for AmF and −20.5 ppm for NaF). Similar variability values were found in the cheek (T0-T1: median 10.09 ppm for AmF and 8.89 ppm for NaF; T1-T2: −9.71 ppm for AmF and −7.63 ppm for NaF). The lowest variability was found in the biofilm of the oral floor (T0-T1: median 0.11 ppm for AmF and 0.13 ppm for NaF; T1-T2: 0.01 ppm for AmF and −0.31 for NaF). All data regarding fluoride variability are summarized in table 3 for AmF and table 4 for NaF. There were no significant differences between the fluoride concentration changes in all oral surfaces from T2 to T3 (Tables 3 and 4).

## Discussion

The histological analysis of tongue and palatal surface in the present study revealed a keratinized epithelium. This is of importance because the tongue and palate have sliding contacts that influence the tribology of their surfaces. The tribological process between tongue and palate is very complex because it simultaneously involves mechanical friction, wear mechanisms, chemical effects and material transfer^42^. The micro- and macro-mechanical tribological mechanisms explain the origin of the tongue coating and its composition. Summarizing these data may explain the mechanism of the natural clearance of the tongue surface and the origin, function, content and amount of the tongue coating compared to other oral biofilms.

Cellular surfaces may be electrically charged, and fluorides may bind to these charged surfaces. As a consequence, this binding may influence fluoride bioavailability, which is considered essential for enamel remineralization^43^.

Two basic types of fluoride reservoirs can persistently increase fluoride concentrations in the fluids surrounding the sites of de- and remineralization of the teeth: the mineral deposits of calcium fluoride (CaF_2_) and fluorapatite and biologically/bacterially bound fluoride deposits^44^. Biologically and bacterially bound fluoride deposits are found in supernatant saliva^37^, salivary sediment^37^,^38^,^39^, and plaque^38^, However, in the available literature, no information was found regarding the fluoride concentration in the different oral biofilms. Therefore, in the present study, the fluoride concentration in the different compartments of the oral cavity was investigated.

The results of this study revealed that, at baseline, the fluoride concentration in the oral biofilm is similar in the cheek and oral floor. Meanwhile, the tongue coating had the lowest and the palate had the highest fluoride values for the four investigated oral surfaces. This finding reflected that the palatal biofilm had the highest affinity towards fluoride ions, followed by the mouth floor, cheek biofilm and the tongue coating.

Tooth brushing with toothpaste containing either AmF or NaF resulted in a significant increase in the fluoride concentration in all oral surface biofilms. These results are in concordance with earlier, similar investigations on AmF toothpaste^33^,^41^.

The tongue biofilm showed the highest fluoride turnover, followed by cheek, palate biofilm and the oral floor after tooth brushing with toothpaste containing either AmF or NaF. The oral floor showed almost no fluctuation in fluoride content after tooth brushing with the NaF toothpaste and the lowest increase after the AmF toothpaste compared to the other oral surfaces. Tooth brushing stimulates the salivary flow rate and results in an increased salivary clearance^45^ of the fluoride from the mouth floor biofilm.

The tongue coating and cheek biofilm showed initially higher affinity towards fluoride ions after tooth brushing with NaF toothpaste compared with AmF toothpaste. This effect was followed by a quick decrease to baseline values at 120 minutes after tooth brushing in the NaF group, while the fluoride concentration in the AmF group remained stable and did not reach baseline values until the end of the study protocol.

The palatal biofilm showed a similar affinity towards fluoride ions after tooth brushing with either the AmF toothpaste or the NaF toothpaste. Fluoride concentration returned to baseline values at 30 minutes after tooth brushing. With regard to the baseline fluoride values of the palatal biofilm, it might be concluded that the palatal biofilm is a fluoride reservoir together with the plaque^41^ and salivary sediment^39^,^41^. However, the total surface of the tongue is more than ⅓ of the total surface area of the oral cavity^20^. Therefore, it might be assumed that the tongue coating, palate biofilm, plaque and sediment serve as the four essential biological/bacterial fluoride reservoirs in the oral cavity. Thus, the fluoride retention in the oral biofilms is an important prerequisite for fluoride bioavailability in the oral cavity. Obviously, the different epithelial sites of the oral cavity contribute differently to fluoride retention. With regard to fluoride bioavailability for caries prophylaxis it has been shown that already small amounts of fluoride are enhancing enamel remineralization^46^,^47^. Although the bioavailable fluoride concentration decreases considerably after application of fluoride, a constant minimal level of fluoride seems to be enough for caries prevention. Fluoride retention the biological surfaces of the oral cavity might help to maintain a constant fluoride bioavailability.

In addition to earlier findings, which demonstrated that the organic components of the salivary sediment bind large amounts of fluoride^41^, this study supports the hypothesis that AmF in particular binds to organic surfaces of oral mucosa because it has surfactant properties, with a lipophilic and a hydrophilic end. This might explain the finding that clearance of fluoride from AmF in the oral cavity is slower than that from NaF, which results in a prolonged bioavailability of fluoride after AmF uptake. This is in accordance with other studies, which also demonstrated a fast clearance of fluoride after NaF application^35^,^36^,^48^,^49^.

As soon as NaF is dissolved in saliva, the NaF dissociates and fluoride becomes chemically reactive. Fluoride is released more slowly from AmF, which results in a prolonged bioavailability. This might explain the extreme changes in fluoride concentration within 120 minutes after NaF application. For future application of fluorides, their distribution and the retention within the oral biofilm should be taken into consideration.

## Conclusions

The results are in accordance with previous studies that showed a dramatic increase in fluoride concentration after tooth brushing in the supernatant saliva, salivary sediment and plaque, followed by a decrease. The fluoride concentration after AmF application remained at a twofold higher level compared with the baseline in the tongue coating and in the palatal biofilm. This may indicate that the tongue coating and the palatal biofilm are the reservoirs for the oral fluoride bioavailability. AmF and NaF have different affinities for the distinct areas of the oral cavity. The results also demonstrate an unequal fluoride distribution in the oral cavity. Thus, the null hypothesis that assumed no differences in the fluoride bioavailability between the different oral areas and between the different fluoride compounds has been rejected.

## Additional Information

**How to cite this article**: Naumova, E. A. *et al.* Dynamics of Fluoride Bioavailability in the Biofilms of Different Oral Surfaces after Amine Fluoride and Sodium Fluoride Application. *Sci. Rep.* **6**, 18729; doi: 10.1038/srep18729 (2016).

## Figures and Tables

**Table 1:** Descriptive statistical data of the AUC after AmF administration.

	Tongue	Palate	Mouth floor	Cheek
Median	18.63	26.82	6.36	18.35
Minimum	5.43	6.61	0.44	2.74
Maximum	56.7	85.89	41.07	66.45
Range	51.27	80.28	40.36	63.71

Values are in ppm.

**Table 2:** Descriptive statistical data of the variability of the fluoride concentration after AmF administration.

	Tongue			Palate			Mouth floor			Cheek		
	T1 − T0	T2 − T1	T3 − T2	T1 − T0	T2 − T1	T3 − T2	T1 − T0	T2 − T1	T3 − T2	T1 − T0	T2 − T1	T3 − T2
Median	14.69	−14.70	−11.00	7.44	−7.34	−0.30	0.11	0.01	−0.07	10.09	−9.71	−0.62
Minimum	3.97	−50.63	−0.30	−23.05	−50.89	−17.02	−21.73	−5.31	−17.35	0.70	−63.42	−16.99
Maximum	51.25	**−**1.55	1.14	51.36	14.80	39.08	7.31	16.41	5.91	57.56	12.33	9.14
Range	47.28	49.07	3.59	74.42	65.70	56.11	29.04	21.73	23.26	60.93	75.76	26.14

Values are in ppm.

**Table 3:** Descriptive statistical data of the variability of the fluoride concentration after NaF administration.

	Tongue			Palate			Mouth floor			Cheek		
	T1 − T0	T2 − T1	T3 − T2	T1 − T0	T2 − T1	T3 − T2	T1 − T0	T2 − T1	T3 − T2	T1 − T0	T2 − T1	T3 − T2
Median	30.43	−28.14	−1.31	19.03	−20.50	−0.35	0.13	−31.00	0.16	8.89	−7.63	−0.57
Minimum	−0.27	−108.69	−8.45	0.63	−59.39	−13.45	−3.51	−15.52	−3.05	−3.37	−53.90	−21.13
Maximum	114.11	−3.66	14.98	64.19	6.20	11.75	16.55	2.09	6.20	57.56	17.76	7.37
Range	114.38	105.02	23.44	63.55	65.60	25.21	20.06	17.62	9.26	60.93	71.66	28.51

Values are in ppm.

**Table 4:** Descriptive statistical data of the AUC after NaF administration.

	Tongue	Palate	Mouth floor	Cheek
Median	33.56	33.79	5.09	16.68
Minimum	7.16	11.25	1.38	4.10
Maximum	121.91	76.52	20.53	65.99
Range	114.75	65.27	19.15	61.89

Values are in ppm.
